# Optimizing the Extraction of Anti-tumor Polysaccharides from the Fruit of *Capparis spionosa* L. by Response Surface Methodology

**DOI:** 10.3390/molecules17067323

**Published:** 2012-06-14

**Authors:** Yu-Bin Ji, Fang Dong, Dong-Bin Ma, Jing Miao, Li-Na Jin, Zhen-Feng Liu, Ling-Wen Zhang

**Affiliations:** 1 Research Center on Life Sciences and Environmental Sciences, Harbin University of Commerce, Harbin 150076, China; Email: dong1234fang@126.com (F.D.); huawuxinde@163.com (Z.-F.L.); lingwen2008@163.com (L.-W.Z.); 2 Engineering Research Center of Natural Anticancer Drugs, Ministry of Education, Harbin 150076, China; Email: madongbin1987@163.com (D.-B.M.); miaojing586@163.com (J.M.); kinglena@126.com (L.-N.J.)

**Keywords:** polysaccharides of *Capparis spionosa* L. (CSPS), extraction optimization, response surface methodology, anti-tumor activity

## Abstract

*Capparis spionosa* L. is a traditional medicinal plant in China and central Asia. In this study, an experiment was designed to investigate the optimization of the extraction of anti-tumor polysaccharides from the fruit of *Capparis spionosa* L. (CSPS) by response surface methodology (RSM). Four independent variables (extraction temperature, extraction time, ratio of water to sample and extraction cycles) were explored. Meanwhile, the *in vivo* anti-tumor activity of CSPS was investigated. The results showed that the experimental data could be fitted to a second-order polynomial equation using multiple regression analysis. The optimum extraction conditions were as follows: extraction temperature 92 °C, extraction time 140 min, ratio of water to sample 26 mL/g, and three extraction cycle. Under these conditions, the yield of polysaccharides reached 13.01%, which was comparable to the predicted yield (12.94%, *p *> 0.05). This indicated that the model was adequate for the extraction process. Additionally, CSPS could prolong the survival time of H_22_ bearing mice *in vivo*. The anti-tumor activities of CSPS were dose-dependent.

## 1. Introduction

In recent years, more and more plant polysaccharides have been used in medicine and health-care food. Plant polysaccharides have various biological activities, such as antioxidant [1,2], free radical scavenging [3], immunostimulatory [4,5], and anti-virus activity [6]. At the same time, a growing amount of research has shown that polysaccharides could resist tumors by improving the immune system and inducing tumor apoptosis [7,8,9,10].

*Capparis spionosa* L., which cultivated in China and central Asia, is a traditional medicinal plant [11,12,13,14,15]. Abundant research suggests that *Capparis spionosa* L. contains a variety of active ingredients, such as volatile oil, sugar ligands, glucose isothiocyanates, alkaloids, and so on [16].

Response surface methodology (RSM) that applies to optimizing conditions in food and pharmaceutical research is an effective tool for optimizing extraction processes [17,18,19]. As one type of RSM, Box-Behnken design (BBD) is popularly use, principally to optimize technical parameters. BBD is prevalent to other approaches required in optimizing a process, such as saving materials, decreasing expenses, reducing time, and so on [20].

Up to now, no detailed investigation has been conducted on optimization of polysaccharide extraction from the fruit of *Capparis spionosa* L. In addition, there are no experiments to explore the anti-tumor activity of CSPS. Therefore, the purpose of the study was to employ a BBD (four factors and three levels) to optimize the effects of extraction temperature, extraction time, ratio of water to sample, and extraction cycles on the yield of polysaccharides obtained from the fruit of *Capparis spionosa* L. (CSPS). Furthermore, the anti-tumor activity of CSPS was evaluated in the search for high quality biological functional principles for use in the pharmaceutical industry.

## 2. Results and Discussion

### 2.1. Effect of Extraction Temperature on the Yield of CSPS

**Figure 1:**
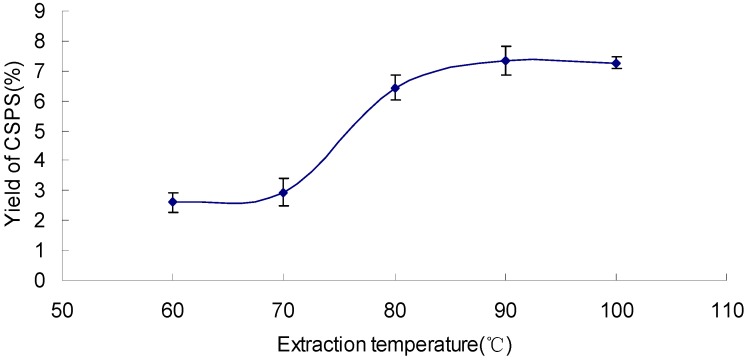
Effect of extraction temperature on the yield of CSPS under extraction time 60 min, ratio of water to sample 10 mL/g, and extraction cycle 1.

The effect of different extraction temperatures (60, 70, 80, 90 and 100 °C) on the yield of CSPS is shown in Figure 1, which indicates that the yield increased with the increasing extraction temperature and reached the maximum value of 7.33% at 90 °C. Then, there was no increase when the extraction temperature at 100 °C. Therefore, 80~100 °C were adopted for the extraction.

### 2.2. Effect of Extraction Time on the Yield of CSPS

**Figure 2:**
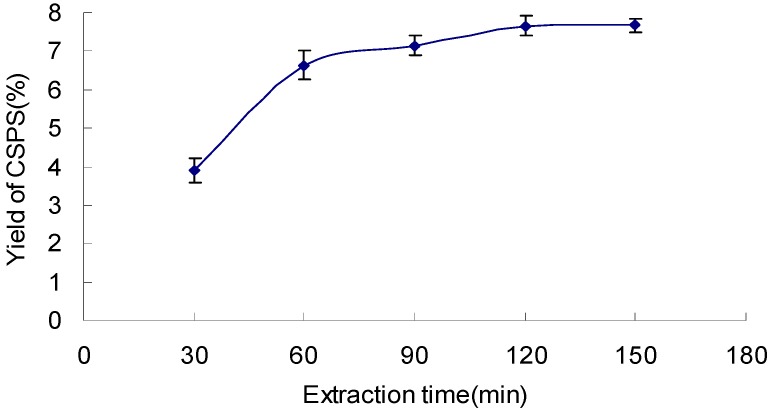
Effect of extraction time on the yield of CSPS under extraction temperature 90 °C, ratio of water to sample 10 mL/g, and extraction cycle 1.

The results shown in Figure 2 maintained a mild slope after 90 min. The yield of CSPS reached a maximum value of 7.66 when the extraction time was 120 min, consequently 90~150 min was considered to be optimal extraction time in the experiment.

### 2.3. Effect of Ratio of Water to Sample on the Yield of CSPS

**Figure 3:**
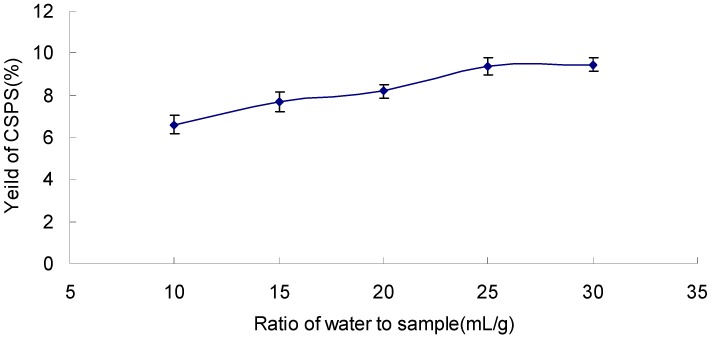
Effect of ratio of water to sample on the yield of CSPS under extraction temperature 90 °C, extraction time120 min, and extraction cycle 1.

As seen from Figure 3, the yield of CSPS reached the critical value 9.8 at the ratio of 25 mL/g, and then started to maintain a dynamic equilibrium. Therefore, the range of water to sample ratios of 20~30 mL/g was used in the present work.

### 2.4. Effect of Extraction Cycles on the Yield of CSPS

**Figure 4:**
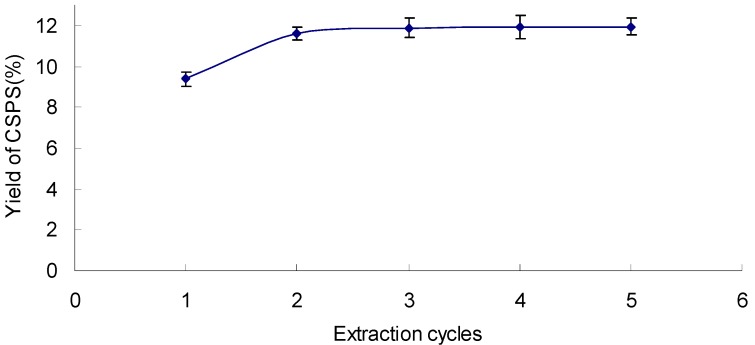
Effect of extraction cycles on the yield of CSPS under extraction temperature 90 °C, extraction time120 min, and ratio of water to sample 25 mL/g.

The result indicated that the yield of CSPS increased with the number of extraction cycles, but there was no significant increase after 2 cycles (Figure 4). So, an extraction cycles range of 1~3 was adopted.

### 2.5. Results of the Yield of CSPS of Optimization of the Procedure

The twenty nine experimental points for optimizing the four individual parameters in the BBD were shown in Table 1. Five replicates (exp. No. 25~29) at the center of the design were used to allow for estimation of a pure error sum of squares. The response value in each trial was the average of triplicates.

**Table 1:** Box-Behnken design matrix of four variables and the experimental observed responses.

No.	X_1_/Extraction temperature (°C)	X_2_/Extraction time (min)	X_3_/Ratio of water to sample (mL/g)	X_4_/Extraction cycles	Yield of CSPS (%)
1	−1(80)	−1(90)	0(25)	0(2)	9.028
2	1(100)	−1(90)	0(25)	0(2)	11.584
3	−1(80)	1(150)	0(25)	0(2)	11.370
4	1(100)	1(150)	0(25)	0(2)	11.458
5	0(90)	0(120)	−1(20)	−1(1)	8.206
6	0(90)	0(120)	1(30)	−1(1)	9.466
7	0(90)	0(120)	−1(20)	1(3)	10.962
8	0(90)	0(120)	1(30)	1(3)	12.190
9	−1(80)	0(120)	0(25)	−1(1)	7.052
10	1(100)	0(120)	0(25)	−1(1)	9.150
11	−1(80)	0(120)	0(25)	1(3)	10.214
12	1(100)	0(120)	0(25)	1(3)	11.792
13	0(90)	−1(90)	−1(20)	0(2)	8.566
14	0(90)	1(150)	−1(20)	0(2)	10.826
15	0(90)	−1(90)	1(30)	0(2)	10.250
16	0(90)	1(150)	1(30)	0(2)	11.090
17	0(80)	0(120)	−1(20)	0(2)	9.722
18	1(100)	0(120)	−1(20)	0(2)	10.968
19	−1(80)	0(120)	1(30)	0(2)	10.202
20	1(100)	0(120)	1(30)	0(2)	11.560
21	0(90)	−1(90)	0(25)	−1(1)	8.426
22	0(90)	1(150)	0(25)	−1(1)	8.568
23	0(90)	−1(90)	0(25)	1(3)	11.002
24	0(90)	1(150)	0(25)	1(3)	12.49
25	0(90)	0(120)	0(25)	0(2)	11.702
26	0(90)	0(120)	0(25)	0(2)	11.547
27	0(90)	0(120)	0(25)	0(2)	11.626
28	0(90)	0(120)	0(25)	0(2)	11.315
29	0(90)	0(120)	0(25)	0(2)	11.918

### 2.6. Model Fitting and Statistical Significance Analysis

By applying multiple regression analysis on the experimental data, the response variable and the test variables were related by the following second-order polynomial equation:


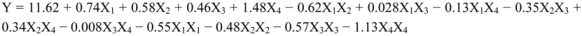


The determination coefficient (R^2^ = 0.9460) is shown in Table 2, indicating that the model could explain 94.60% of the response value changes and only 5.40% of the total variations was not explained by the model. At the same time, a low value 0.0439 of coefficient of the variation (C.V.) indicated a high degree of precision and a good deal of reliability of the experimental value. This meant that the model could be used to analyze and predict polysaccharide extraction process results.

**Table 2:** Test result of significance for regression coefficients.

Parameter	Estimate	df	Standard error	95%CI		*F*-value	*p*-value
				Low	High		
intercept	11.62	1	0.21	11.18	12.06		
X_1_	0.74	1	0.13	0.46	1.03	31.35	<0.0001
X_2_	0.58	1	0.13	0.29	0.86	19.00	0.0007
X_3_	0.46	1	0.13	0.17	0.74	11.94	0.0039
X_4_	1.84	1	0.13	1.20	1.77	124.09	<0.0001
X_1_X_2_	−0.62	1	0.23	−1.11	−0.12	7.91	0.0179
X_1_X_3_	0.028	1	0.23	−0.47	0.52	0.015	0.9048
X_1_X_4_	−0.13	1	0.23	−0.62	0.36	0.32	0.5809
X_2_X_3_	−0.35	1	0.23	−0.85	0.14	2.83	0.1451
X_2_X_4_	0.34	1	0.23	−0.16	0.83	2.14	0.1656
X_3_X_4_	−0.008	1	0.23	−0.50	0.49	0.005	0.9727
X_1_X_1_	−0.55	1	0.18	−0.94	−0.17	9.41	0.0083
intercept	11.62	1	0.21	11.18	12.06		
X_2_X_2_	−0.48	1	0.18	−0.87	−0.097	7.19	0.0179
X_3_X_3_	−0.57	1	0.18	−0.95	−0.18	9.81	0.0074
X_4_X_4_	−1.13	1	0.18	−1.51	−0.74	38.92	<0.0001

Note: R^2^ = 0.9460, Adjusted R^2^ = 0.8921, C.V. = 0.0439.

The *p*-value is used as an important tool to check the significance of each coefficient, which in turn may indicate the pattern of the interactions between the variables. The smaller the value of the *p*-value, the more significant the corresponding coefficient was. As seen from Table 2, the linear coefficients (X_1_, X_2_, X_3_, X_4_), cross product coefficient (X_1_X_2_) and quadratic coefficients (X_1_^2^, X_2_^2^, X_3_^2^, X_4_^2^) of the model were significant, with a very small *p*-value (*p *< 0.05). The other term coefficients were not significant (*p *> 0.05).At the same time, *F*-value and *p*-value (*F *= 17.53, *p *< 0.0001) of the model, which are shown in Table 3, indicated that the regression model was very significant. Lack of fit (*F *= 5.71, *p *> 0.05) was not significant.

**Table 3:** Analysis of variance for fitted quadratic polynomial model.

Source	Sum of squares	df	Mean square	*F*-value	*p*-value	
Model	51.94	14	3.71	17.53	<0.0001	significant
Residual	2.96	14	0.21			
Lack of fit	2.77	10	0.28	5.71	0.0583	
Pure error	0.19	4	0.048			
Cor total	54.90	28				

### 2.7. Optimization of Extraction Conditions of CSPS

**Figure 5:**
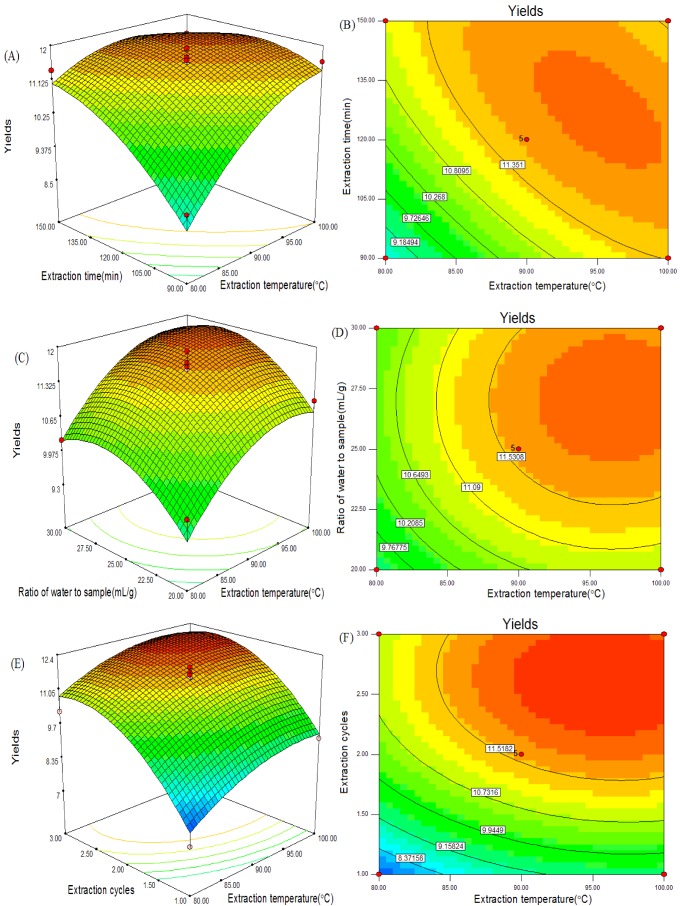

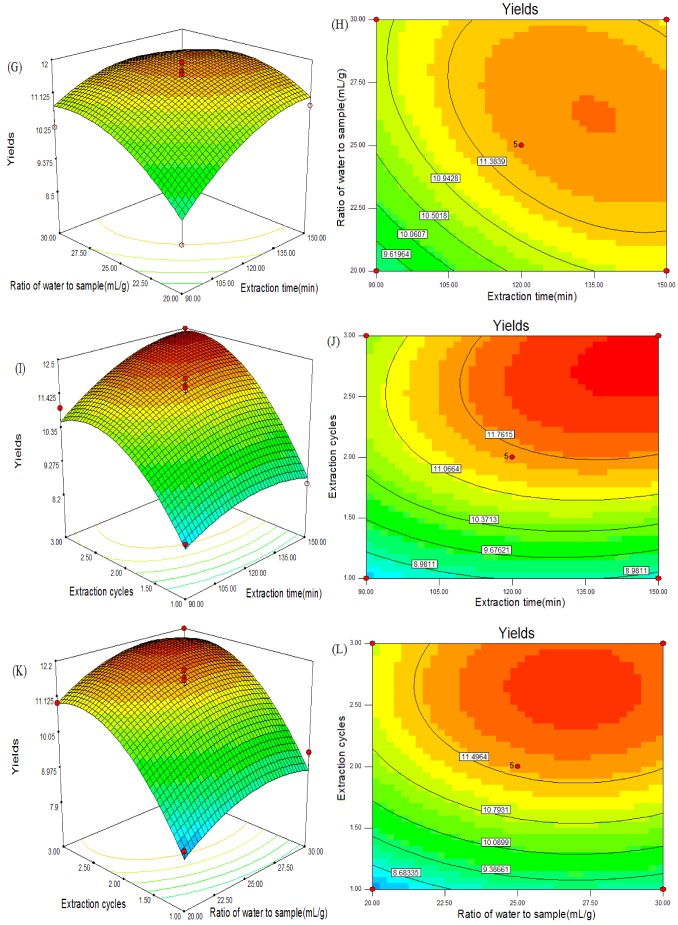
Response surface plots (3-D) and contour plots (2-D) showing the effects of variables (X_1_: extraction temperature; X_2_: extraction time; X_3_: ratio of water to sample; X_4_: extraction cycles).

Based on the regression equation obtained by Design-Expert 7.0, the graphical representations of the yield of CSPS affected by X_1_, X_2_, X_3_ and X_4_ are presented in Figure 5. The independent variables and maximum predicted values from the figures corresponded with the optimum values of the dependent variables obtained by the equation.

The contour plots can reflect the strength of the interaction effects. When the contour plots are oval, it means that the interaction of two independent variables is significant. In contrast, the round contour plots are considered not significant [21,22]. According to Figure 5 and Table 2, the interaction between extraction temperature and extraction time was significant (*p *< 0.05).

The optimal extraction conditions were obtained from response surface analysis as follows: extraction temperature 92.46 °C, extraction time 138.53 min, ratio of water to sample 26.06 mL/g, extraction cycles 2.74. The maximum predicted yield of CSPS was 12.94%.

### 2.8. Verification of Predictive Model

It is desirable to test the accuracy and reliability of the model equation for predicting an optimum response. Taking fully into account the actual operating convenience, the confirmatory experimental verification was tested under the conditions: extraction temperature 92 °C, extraction time 140 min, the ratio of water to sample 26 mL/g, and extraction cycles 3. A mean value of (13.01 ± 0.08) % (n = 3) was obtained from the confirmatory experiment. The difference between the real value and the predicted value was not significant (*p *> 0.05). It indicated that the model was adequate for the extraction process (Table 4).

**Table 4:** Predicted and experimental values of the responses at optimum conditions.

	Extraction temperature (°C)	Extraction time (min)	Ratio of water to sample (mL/g)	Extraction cycles	Yield of CSPS (%)
Predicted optimum condition	92.46	138	26.06	2.74	12.94
Experimental optimum condition	92	140	26	3	13.01 ± 0.08 *^a^

* means ± standard deviations (n = 3), ^a^ compared with predicted yield, *p *> 0.05.

### 2.9. Anti-Tumor Activity of CSPS *in Vivo*

Life prolonging experiments are used to detect whether a sample has anti-tumor activity [23,24]. The size of the values reflect the strength of any anti-tumor activity. As seen from Table 5, the survival times of the CSPS groups were prolonged significantly in a dose-dependent manner and the rate of life prolongation of the mid-dose CSPS group was better than that of the positive group for the same dosing. The difference of survival time in mice between the low-dose CSPS group and control group was significant (*p *< 0.05). The survival time in control group mice was significantly extremely lower than that of the mid-dose CSPS group (*p *< 0.01, Table 5), and the same happened between the control and high-dose CSPS groups. The results showed that CSPS had *in vivo* anti-tumor activity by prolonging the survival time of H_22_ bearing mice.

**Table 5:** Effects of CSPS on survival time of tumor H_22_ bearing mice.

Groups	Number	Dose (mg/kg)	Survival time (d)	Prolonging rate (%)
Control	12	Normal saline	10.24 ± 2.97	-
Low-CSPS	12	50	12.66 ± 2.53 *	23.63
Mid-CSPS	12	100	15.48 ± 3.15 **	51.17
High-CSPS	12	200	16.72 ± 2.31 **	63.28
APS	12	100	14.89 ± 2.35 **	45.41

Compared with control group, * *p *< 0.05 and ** *p *< 0.01. Data were expressed as means ± standard deviations (n = 10).

## 3. Experimental

### 3.1. Materials

The fruit of *Capparis spionosa* L. was purchased from Xinjiang Wanbang Biotechnology Co. Ltd. (Urumqi, China) Ethanol, dehydrated alcohol, acetone and benzine (analytical grade) were purchased from Tianjin FuChen Chemical Reagent Factory (Tianjin, China). Normal saline was purchased from Harbin Triple Pharmaceutical Co., Ltd. (Harbin, China). Astragalus polysaccharide (APS) was obtained from the Institute of Pharmacology, Harbin University of Commerce (Harbin, China).

### 3.2. Extraction and Yield of CSPS

The dried fruit of *Capparis spionosa* L. (2,500 g) was ground in a blender to obtain a fine powder. The powder was extracted with 95% ethanol (5,000 mL, ×3 cycles) at 90 °C for 3 h, and filtered through nylon cloth (pore diameter 38 μm). The residue was dried under reduced pressure. Each dried residue sample (10 g) was extracted by a water bath under a set of designed temperature, extraction time, water-sample ratio and extraction cycle conditions. The extraction solution was separated from insoluble residue by centrifugation (4,000 × g for 5 min, at 20 °C), and then precipitated by dehydrated alcohol to a final concentration of 80% (v/v). The precipitate (CSPS) collected by centrifuge (4,000 × g for 10 min, at 20 °C) was washed with dehydrated alcohol, acetone and benzine, and vacuum dried. The yield of CSPS was calculated according to the following equation:


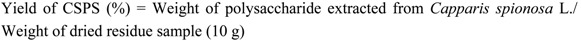


### 3.3. Optimization Design

On the basis of single-factor experiments for the polysaccharide production, suitable ranges of extraction temperature, extraction time, ratio of water to sample, extraction cycles were preliminarily determined. A BBD with four independent variables (X_1_, extraction temperature; X_2_, extraction time; X_3_, ratio of water to sample; X_4_, extraction cycles) at three levels was performed. The range of independent variables and their levels is shown in Table 6. The yield of CSPS was the dependent variable.

**Table 6:** Independent variables and their levels used in the response surface design.

Independent variables		Factor level	
	−1	0	1
X_1_ extraction temperature (°C)	80	90	100
X_2_ extraction time (min)	90	120	150
X_3_ ratio of water to sample (mL/g)	20	25	30
X_4_ extraction cycles	1	2	3

### 3.4. *In Vivo* Life Prolonging Experiments

Experimental animal models: Kun ming mice were transplanted ascites tumor H_22_ 0.2 mL (4 × 10^6^ cells/mL) in the abdominal cavity.

Groups: The weighed mice after inoculation were randomly divided into six groups (n = 12), half male and half female. Control group: Mice were treated with normal saline [100 mg/(kg·d)]. Positive group: mice were treated with Astragalus polysaccharide (APS) at a dosage of 100 mg/(kg·d). CSPS groups: Mice of low, middle, high-dose groups were treated with CSPS at a dosage of 50, 100, 200 mg/(kg·d), respectively.

After 24 h of inoculation, the mice bearing tumor H_22_ were treated in gavage (i.g.) according to the above groups for 7 days. Then, the survival time of mice was recorded. The extension rate was calculated by the following formula:


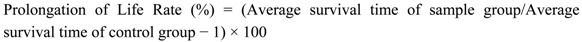


### 3.5. Statistical Analysis

Design-Expert (Version 7.0) software was used to analyze the experimental data. Statistical comparison within groups was carried out by one way ANOVA. A *p*-value of less than 0.05 was considered to be significant statistically. All determinations were carried out in triplicate.

## 4. Conclusions

In this paper, the extraction conditions for CSPS were optimized by BBD, and a quadratic polynomial model was obtained from RMS. The confirmatory experimental optimum conditions of CSPS were as follows: extraction temperature 92 °C, extraction time 140 min, the ratio of water to sample 26 mL/g, and extraction cycles 3. The optimal yield of (13.01 ± 0.09) % which was obtained from confirmatory experiments closely matched the predicted yield of 12.94%. Additionally, CSPS could prolong the survival time of H_22_ bearing mice in a dose-dependent manner.

Simultaneously, our previous research has indicated that CSPS could inhibit the proliferation of human hepatoma HepG2 cells (IC_50_ = 471.53 μg/mL) and induce HepG2 cells to apoptosis *in vitro*. Under the laser scanning confocal microscope, we observed that the cytoplasm was leaked from un-intact HepG2 cells and the cells stained with AO/EB were disrupted to pieces in mid-CSPS and high-CSPS group [25]. This data, combined with that of the present experiments, shows that CSPS has potential anti-tumor capacity. Further research on the chemical structure and anti-tumor mechanism of CSPS will be carried out in the future.
